# Assessment of the toxicity and carcinogenicity of double-walled carbon nanotubes in the rat lung after intratracheal instillation: a two-year study

**DOI:** 10.1186/s12989-022-00469-8

**Published:** 2022-04-22

**Authors:** Dina Mourad Saleh, Shengyong Luo, Omnia Hosny Mohamed Ahmed, David B. Alexander, William T. Alexander, Sivagami Gunasekaran, Ahmed M. El-Gazzar, Mohamed Abdelgied, Takamasa Numano, Hiroshi Takase, Makoto Ohnishi, Susumu Tomono, Randa Hussein Abd el Hady, Katsumi Fukamachi, Jun Kanno, Akihiko Hirose, Jiegou Xu, Shugo Suzuki, Aya Naiki-Ito, Satoru Takahashi, Hiroyuki Tsuda

**Affiliations:** 1 Nanotoxicology Lab Project, Nagoya City University, 3-1 Tanabe-Dohri, Mizuho-ku, Nagoya, 467-8603 Japan; 2 Department of Experimental Pathology and Tumor Biology, Graduate School of Medical Sciences, Nagoya City University, Nagoya, Japan; 3 Department of Forensic Medicine and Clinical Toxicology, Faculty of Medicine, Assuit University, Assuit, Egypt; 4 College of Basic Medical Sciences, Anhui Medical University, Hefei, China; 5 Department of Forensic Medicine and Clinical Toxicology, Faculty of Medicine, Aswan University, Aswan, Egypt; 6 Department of Forensic Medicine and Toxicology, Faculty of Veterinary Medicine, Alexandria University, Alexandria, Egypt; 7 Department of Forensic Medicine and Toxicology, Faculty of Veterinary Medicine, Beni-Suef University, Beni-Suef, Egypt; 8 Department of Pediatrics and Human Development, Michigan State University, Michigan, USA; 9 Core Laboratory, Graduate School of Medicine, Nagoya City University, Nagoya, Japan; 10 Japan Industrial Safety and Health Association, Japan Bioassay Research Center, Hadano, Kanagawa Japan; 11 Department of Microbiology and Immunology, Aichi Medical University School of Medicine, Nagakute, Japan; 12 Department of Neurotoxicology, Graduate School of Medical Sciences, Nagoya City University, Nagoya, Japan; 13 National Institute Hygienic Sciences, Kawasaki, Japan; 14 Department of Immunology, School of Basic Medical Sciences, Anhui Medical University, Hefei, China; 15 Department of Molecular Pathology, Osaka Metropolitan University Graduate School of Medicine, Osaka, Japan

**Keywords:** Double walled carbon nanotubes, Two-year study, Toxicity, Carcinogenicity, Rats

## Abstract

**Background:**

Considering the expanding industrial applications of carbon nanotubes (CNTs), safety assessment of these materials is far less than needed. Very few long-term in vivo studies have been carried out. This is the first 2-year in vivo study to assess the effects of double walled carbon nanotubes (DWCNTs) in the lung and pleura of rats after pulmonary exposure.

**Methods:**

Rats were divided into six groups: untreated, Vehicle, 3 DWCNT groups (0.12 mg/rat, 0.25 mg/rat and 0.5 mg/rat), and MWCNT-7 (0.5 mg/rat). The test materials were administrated by intratracheal-intrapulmonary spraying (TIPS) every other day for 15 days. Rats were observed without further treatment until sacrifice.

**Results:**

DWCNT were biopersistent in the rat lung and induced marked pulmonary inflammation with a significant increase in macrophage count and levels of the chemotactic cytokines CCL2 and CCL3. In addition, the 0.5 mg DWCNT treated rats had significantly higher pulmonary collagen deposition compared to the vehicle controls. The development of carcinomas in the lungs of rats treated with 0.5 mg DWCNT (4/24) was not quite statistically higher (p = 0.0502) than the vehicle control group (0/25), however, the overall incidence of lung tumor development, bronchiolo-alveolar adenoma and bronchiolo-alveolar carcinoma combined, in the lungs of rats treated with 0.5 mg DWCNT (7/24) was statistically higher (p < 0.05) than the vehicle control group (1/25). Notably, two of the rats treated with DWCNT, one in the 0.25 mg group and one in the 0.5 mg group, developed pleural mesotheliomas. However, both of these lesions developed in the visceral pleura, and unlike the rats administered MWCNT-7, rats administered DWCNT did not have elevated levels of HMGB1 in their pleural lavage fluids. This indicates that the mechanism by which the mesotheliomas that developed in the DWCNT treated rats is not relevant to humans.

**Conclusions:**

Our results demonstrate that the DWCNT fibers we tested are biopersistent in the rat lung and induce chronic inflammation. Rats treated with 0.5 mg DWCNT developed pleural fibrosis and lung tumors. These findings demonstrate that the possibility that at least some types of DWCNTs are fibrogenic and tumorigenic cannot be ignored.

**Supplementary Information:**

The online version contains supplementary material available at 10.1186/s12989-022-00469-8.

## Background

Carbon nanotubes (CNTs) are composed of concentric one-atom thick graphene cylinders and have a wide range of applications [1]. The ability to manipulate the lengths and the number of graphene cylinders that compose CNTs has allowed the production of specific CNTs with different lengths and thicknesses, and these differences result in different CNTs having different physical properties. In general, CNTs are divided into two types single-walled carbon nanotubes (SWCNT) and multi-walled carbon nanotubes (MWCNTs) with double-walled carbon nanotubes (DWCNT) sometimes being considered a distinct class of MWCNT. Multi-walled carbon nanotubes can be divided into two general subtypes, tangled and straight.

The very light weight of CNTs make them easily airborne and inhaled. Consequently, the possibility that CNTS may exhibit foreign body toxicity in the airways and pleura is of concern [2–7]: also see Hansen and Lennquist 2020 [8]. Harmful fibrous particles like asbestos are known to induce fibrosis and cancer through chronic unresolved inflammation characterized by inflammatory cell accumulation and production of reactive oxygen and nitrogen spices (ROS & RNS) that can damage DNA and through repeated cycles of tissue damage and repair fix potentially transforming mutations into daughter cells [9–15].

Despite the widespread production and use of CNTs very few in vivo studies with a duration of 18 months or more have been carried out to assess the toxicity and carcinogenicity of these materials in experimental animals [16–24]. At the time of this writing, IARC has evaluated only MWCNT-7 as carcinogenic in experimental animals and as being possibly carcinogenic in humans (Group 2B). All other CNTs had inadequate evidence in experimental animals for carcinogenicity and they were not classifiable as to their carcinogenicity to humans [11].

We have established that instillation of insoluble test materials, such as CNTs, into the rat lung every other day over the course of 2 weeks (8 doses in total) results in distribution of the test material throughout each of the lung lobes [25]: we refer to this instillation procedure as intratracheal intrapulmonary instillation (TIPS). Using TIPS, we assessed the toxicity and carcinogenicity of MWCNT-N, MWCNT-7, MWCNT-A, and MWCNT-B in long term studies. The thick straight MWCNT-N (40 layers) induced both lung tumors and malignant pleural mesotheliomas [23]; MWCNT-7 (more than 40 layers) induced malignant pleural mesotheliomas [20]; a thin tangled MWCNT, referred to as MWCNT-B, (15 layers) induced lung tumors [22] and MWCNT-A (very thick 150 layers) was shown to be a likely lung carcinogen [22].

However, very little is known about the in vivo toxic effect of DWCNTs. The only available results are short term studies in mice and our short term study in rats. Crouzier et al. found inflammatory reactions in mice 6, 24, and 48 h after a single intranasal instillation of 1.5 mg/kg DWCNT (1.2–3.5 nm diameter, 1–10 μm length) [26]. Tian et al. found that in mice inflammatory lesions were not resolved after a 7 day observation period after a single intratracheal instillation of 50 μg DWCNT (3.5 nm diameter, 1–10 μm length) [27]. Sager et al. reported that mice administered 40 μg DWCNT (1–2 nm diameter, < 5 μm length) by pharyngeal aspiration had developed alveolitis and lung fibrosis 56 days after administration of the DWCNT [28]. O'Shaughnessy et al. exposed mice to DWCNT by whole body inhalation at a dose of 10.8 mg/m^3^, 4 h/day for 5 days. DWCNT caused inflammation and tissue injury to the lung which was resolved 2 weeks after the end of exposure [29]. El-Gazzer et al. administered DWCNT (1–3 nm diameter, due to the tangled nature of the fibers the length could not be measured) and MWCNT-7 (55.5 ± 12 nm diameter, 6.5 ± 2.4 μm length) by TIPS to rats. Six weeks after administration the degree of pulmonary and pleural toxicity induced by MWCNT-7 was much higher than that induced by DWCNT. DWCNT caused more extensive granulation tissue formation encapsulating the fibers than MWCNT-7 [30]. In mice and rats, administration of DWCNT by instillation or pharyngeal aspiration resulted in inflammation that lasted up to 6 weeks (rats) to 8 weeks (mice), while mice exposed to DWCNT by inhalation developed inflammation during exposure, but 2 weeks after the termination of exposure the inflammation had resolved. Based on these findings we conducted the present long-term study to assess the chronic toxicity and carcinogenicity of DWCNT in the rat lung.

## Results

### Interim and terminal sacrifices

Rats were divided into six groups: no treatment (27 rats); rats administered vehicle alone (30 rats); rats administered 0.125 mg DWCNT (30 rats); rats administered 0.25 mg DWCNT (33 rats); rats administered 0.5 mg DWCNT (30 rats); and rats administered 0.5 mg MWCNT-7 (30 rats). One untreated rat died prior to week 52 and was excluded from the study, therefore, the untreated group contained 26 rats. At week 52 an interim sacrifice was performed on 5 rats from the untreated, vehicle, 0.125 mg DWCNT, and 0.5 mg MWCNT-7 group; 7 rats from the 0.25 mg DWCNT group; and 6 rats from the 0.5 mg DWCNT group. Rats found dead after week 52 underwent terminal necropsy and rats found moribund after week 52 and rats surviving to the end of the study underwent terminal sacrifice. The final terminal sacrifice was performed at 104 weeks on rats from the untreated (17 rats), vehicle (17 rats), 0.125 mg DWCNT (18 rats), 0.25 mg DWCNT (20 rats) and 0.5 mg DWCNT (16 rats) groups. In the MWCNT-7 group, 16 rats were found moribund or died due to mesothelioma prior to the end of week 90. Therefore, in order to collect pleural lavage fluid the remaining 9 rats in this group underwent final terminal sacrifice at week 91. In addition, in the sixteen MWCNT-7 treated rats that were found moribund or died due to mesothelioma there was extensive invasion of the lungs by the mesotheliomas, making evaluation of proliferative lesion development (other than mesothelioma) in these rats unreliable. Therefore, only the nine MWCNT-7 treated rats that survived for 91 weeks were evaluated for the parameters shown in Table 3. Throughout this report, terminal sacrifice refers to sacrifices performed after week 52 and final sacrifice refers to the terminal sacrifices performed at weeks 91 and 104.

### Characterization of the test substance

**Fig. 1:**
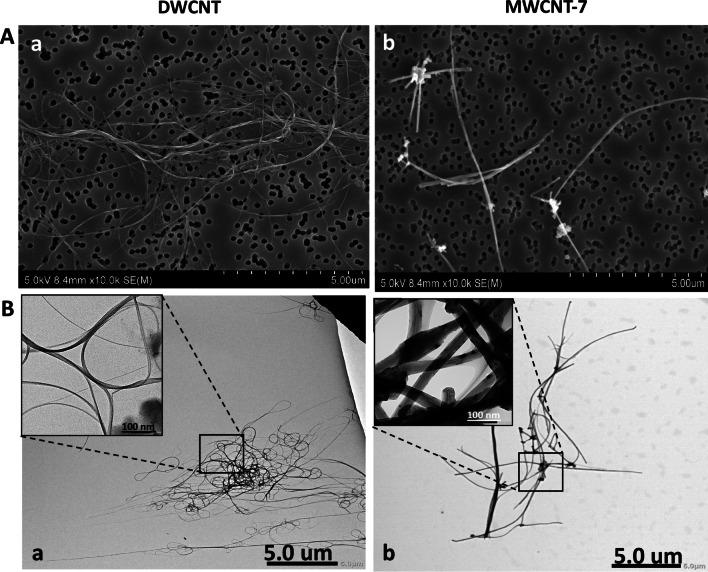
Characterization of test materials in suspension. **A** shows scanning electron microscopy images of (**a**) DWCNT and (**b**) MWCNT-7 fibers and **B** shows transmission electron microscopy images of (**a**) DWCNT and (**b**) MWCNT-7 fibers

Representative SEM and TEM images of DWCNT and MWCNT fibers in suspension, prior to TIPS administration, are shown in Fig. 1, and fiber lengths and diameters are presented in Table 1. DWCNT are thin and tangled and MWCNT are relatively thick and straight. Prior to TIPS administration, DWCNT fibers had a mean diameter of 14.32 ± 10.04 nm, and DWCNT recovered from lung tissue at 104 weeks had a mean diameter of 15.10 ± 13.99 nm. The lengths of the DWCNT fibers could not be measured due to the tangled nature of the fibers. Prior to TIPS administration, MWCNT fibers had a diameter of 76.49 ± 31.14 nm and a mean length of 8.79 ± 4.41 um. MWCNT recovered from lung tissue at 91 weeks had a diameter of 69.42 ± 23.76 nm and a mean length of 8.45 ± 5.29 μm. The size of the DWCNT and MWCNT-7 fibers recovered from the lung tissue at 104 weeks and 91 weeks, respectively, was not significantly different from the size of the fibers in suspension prior to TIPS administration.

**Table 1:** Size of DWCNT and MWCNT-7 fibers in the vehicle before dosing and at weeks 104 and 91

	Length before dosing	Length at 104 & 91 weeks	Diameter before dosing	Diameter at 104 and 91 weeks
DWCNT	NA ^a^	NA ^a^	14.32 + 10.04 nm	15.10 + 13.99 nm
MWCNT-7	8.79 + 4.41 μm	8.45 + 5.29 μm	76.49 + 31.14 nm	69.42 + 23.76 nm

^a^Not available (unable to measure DWCNT fiber length because of agglomerate formation)

### Biodegradation of DWCNT and MWCNT-7 in vitro

DWCNT and MWCNT-7 were added to cultures of RAW cells, a mouse macrophage cell line, or to plates without cells. Plates were then incubated for 5 days. The effective lengths of the DWCNTs incubated without (control) and with RAW cells were estimated using far infrared absorption. The absorption peaks of both samples were observed around 20 cm^−1^ and the peak shift after incubation was within experimental error: this indicates that the changes in effective length between the controls and the DWCNTs incubated with RAW cells were below detectable limits and that the DWCNTs were not measurably damaged by incubation with RAW cells for 5 days (data not shown).

### Macroscopic findings

The lungs of rats treated with 0.125, 0.25, and 0.5 mg DWCNT and 0.5 mg MWCNT-7 showed grayish discoloration throughout all lung lobes. The parabronchial and mediastinal lymph nodes of the DWCNT treated rats were dark gray, and the parabronchial and mediastinal lymph nodes of the MWCNT-7 treated rats were entirely black.

### Incidence of proliferative lesions and survival

No significant differences in the average survival time was observed between the untreated group (102 ± 6 weeks), the vehicle group (99 ± 10 weeks), the 0.125 mg/rat DWCNT group (99 ± 8 weeks), the 0.25 mg/rat DWCNT group (101 ± 7 weeks), and the 0.5 mg/rat DWCNT group (100 ± 4 weeks).

Table 2 shows the incidences of proliferative lesions at 52 weeks. No lung tumors were found in any of the rats sacrificed at 52 week. One rat in the MWCNT-7 group developed a malignant pleural mesothelioma. The incidences of bronchioloalveolar hyperplasia (BAH) were significantly higher in the DWCNT 0.25 mg (5/6) (p < 0.05) and 0.5 mg (6/7) (p < 0.05) groups compared to the vehicle group (0/5). BAH in the DWCNT 0.25 and 0.5 mg groups was also significantly higher (p < 0.05) than in the MWCNT-7 group (1/7).

**Table 2:** Incidence of proliferative and neoplastic lesions at 52 weeks

	Untreated	Vehicle	DWCNT 0.125 mg/rat	DWCNT 0.250 mg/rat	DWCNT 0.500 mg/rat	MWCNT 0.500 mg/rat
Number of rats examined	5	5	5	7	6	5
Bronchiolo-alveolar Hyperplasia (BAH)	0	0	2	5*^,#^	5*^,#^	1
Pleural Mesothelioma	0	0	0	0	0	1

*Difference from Vehicle group: p < 0.05

^#^Difference from MWCNT-7 group: p < 0.05

No lung tumors were found in any of the rats sacrificed at 52 wk

**Fig. 2:**
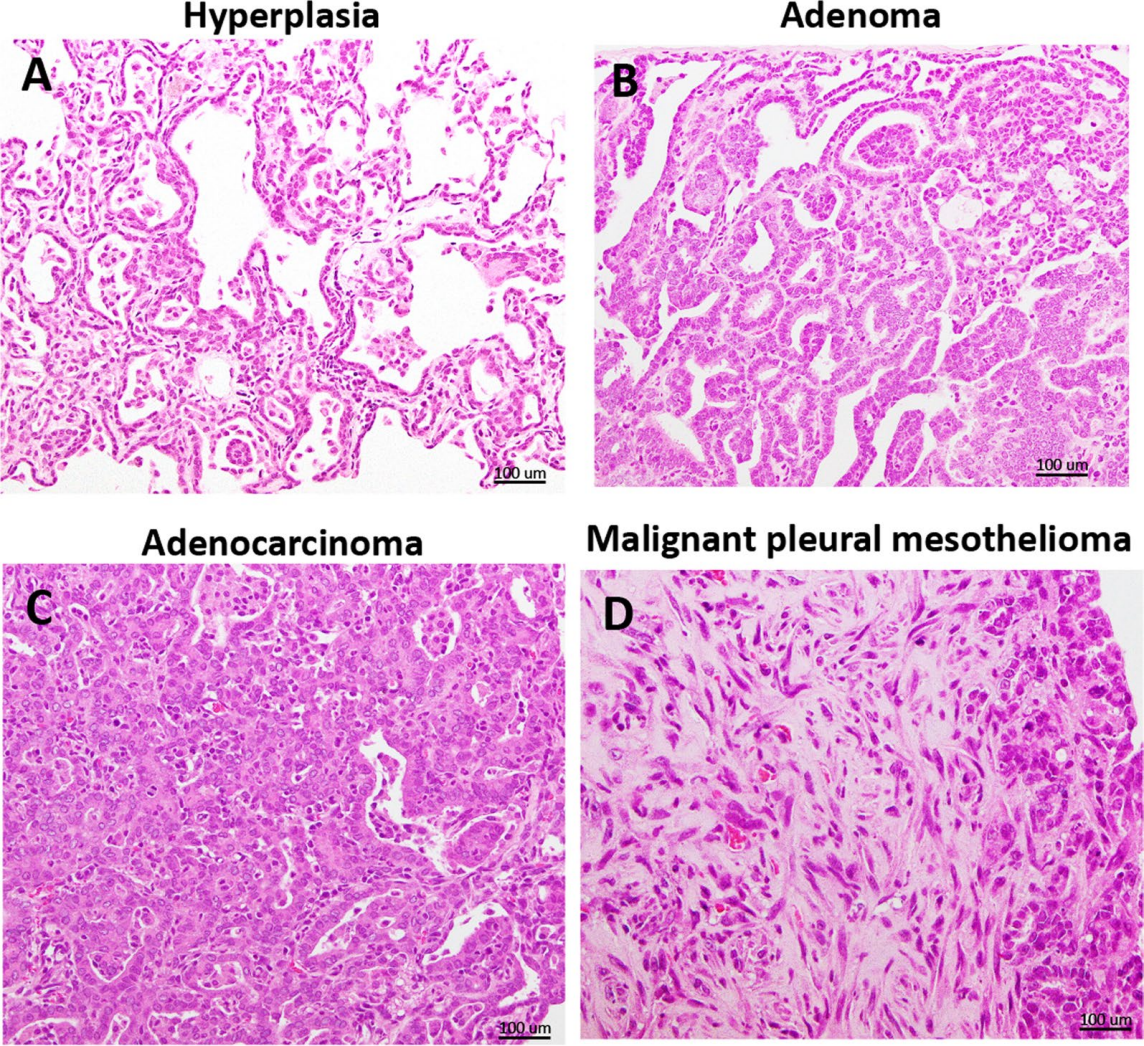
Representative preneoplastic and neoplastic lesions. **A** Bronchiolo-alveolar hyperplasia, **B** Bronchiolo-alveolar adenoma, **C** Bronchiolo-alveolar adenocarcinoma, and **D** Malignant pleural mesothelioma

Table 3 shows the incidences of proliferative lesions that developed in the untreated, vehicle, and DWCNT treated rats that survived beyond 52 weeks and in the MWCNT-7 treated rats that survived for 91 weeks: as noted above, the MWCNT-7 rats that died prior to 91 weeks due to mesotheliomas had extensive invasion of the lungs by the mesotheliomas, making evaluation of proliferative lesions other than mesothelioma in these rats unreliable. Therefore, only the nine MWCNT-7 treated rats that survived for 91 weeks were evaluated for the parameters shown in Table 3. There was an increase in both bronchioloalveolar adenomas (BAA) and bronchioloalveolar carcinomas (BAC) in the DWCNT groups compared with the vehicle control group. However, the development of carcinomas in the lungs of rats treated with 0.5 mg DWCNT (4/24) was not quite statistically higher (p = 0.0502) than the vehicle control group (0/25). On the other hand, the incidence of total lung tumors (BAA + BAC) in the DWCNT 0.5 mg group (7/24) was significantly higher (p < 0.05) than the vehicle group (1/25). At the final sacrifice at 91 weeks, three rats had developed lung tumors (BAA + BAC), which has statistical significance compared to the vehicle control group (1/25). The incidence of malignant pleural mesothelioma in the MWCNT-7 group (16/25) was significantly higher (p < 0.001) than the vehicle group (0/25): this is not shown in Table 3. One rat in the 0.25 mg DWCNT group and one rat in the 0.50 mg DWCNT group also developed mesotheliomas, however, both of these mesotheliomas developed in the visceral pleura and as discussed below are unlikely to be relevant to human mesothelioma development. Two rats, one in the 0.125 mg DWCNT group and one in the 0.50 mg DWCNT group, also developed malignant peritoneal mesotheliomas, however, aged male Fischer 344 rats are prone to developing spontaneous peritoneal mesotheliomas [31]. Figure 2 shows typical lesions that developed in these rats. Other tumors including leukemia, pituitary tumors, mammary tumors, and scrotal malignant mesotheliomas were not treatment related.

**Table 3:** Incidence of proliferative lesions at the terminal sacrifice^a^

	Untreated	Vehicle	DWCNT 0.125 mg/rat	DWCNT 0.250 mg/rat	DWCNT 0.500 mg/rat	MWCNT 0.500 mg/rat
Rats examined	21	25	25	26	24	9^a^
Bronchiolo-alveolar hyperplasia (BAH)	4	4	1	4	0	1
Bronchiolo-alveolar adenoma (BAA)	1	1	3	2	3	1
Bronchiolo-alveolar adenocarcinoma (BAC)	0	0	1	2	4	2
BAA + BAC	1	1	4	4	7*	3*
Malignant pleural mesothelioma	0	0	0	1^b^	1^b^	0
Malignant peritoneal mesothelioma	0	0	1	0	1	0

^a^In the MWCNT-7 group, 16 animals died from malignant mesothelioma after 52 weeks and before 91 weeks. Because of the invasion of the lungs by the mesotheliomas, these animals could not be accessed for development of BAH, BAA, or BAC or the site of mesothelioma development, and therefore, are not included in Table 3

^b^The malignant pleural mesothelioma developed in the visceral pleura

^*^Significantly different from the vehicle group: p < 0.05 and 0.001

### Histopathological evaluation

**Fig. 3:**
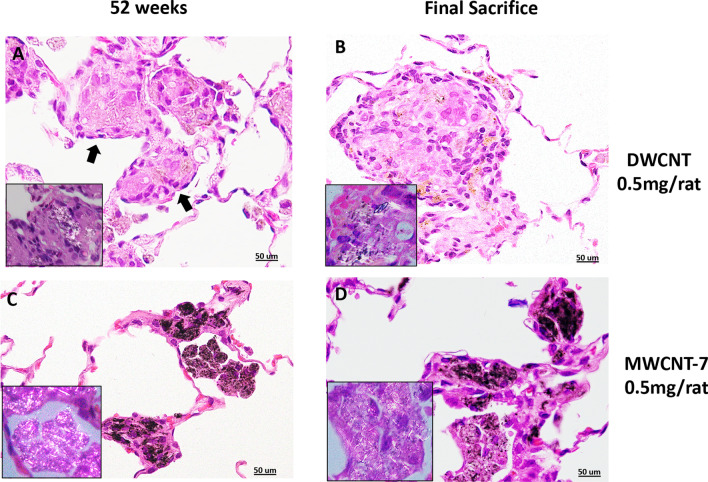
Lung sections of rats administered 0.5 mg DWCNT at 52 weeks (**A**) and 104 weeks (**B**). The majority of the DWCNT is encapsulated in granulation tissue (arrows). The boxed areas are higher magnifications using a polarized lens showing DWCNT fibers. Lung sections of rats administered MWCNT (0.5 mg) at 52 weeks (**C**) and 91 weeks (**D**). MWCNT-7 can be seen encapsulated inside granulation tissue. In contrast to DWCNT, free macrophages phagocytosing MWCNT-7 fibers are a common feature. The boxed areas are higher magnifications using a polarized lens showing MWCNT fibers in free macrophages

**Fig. 4:**
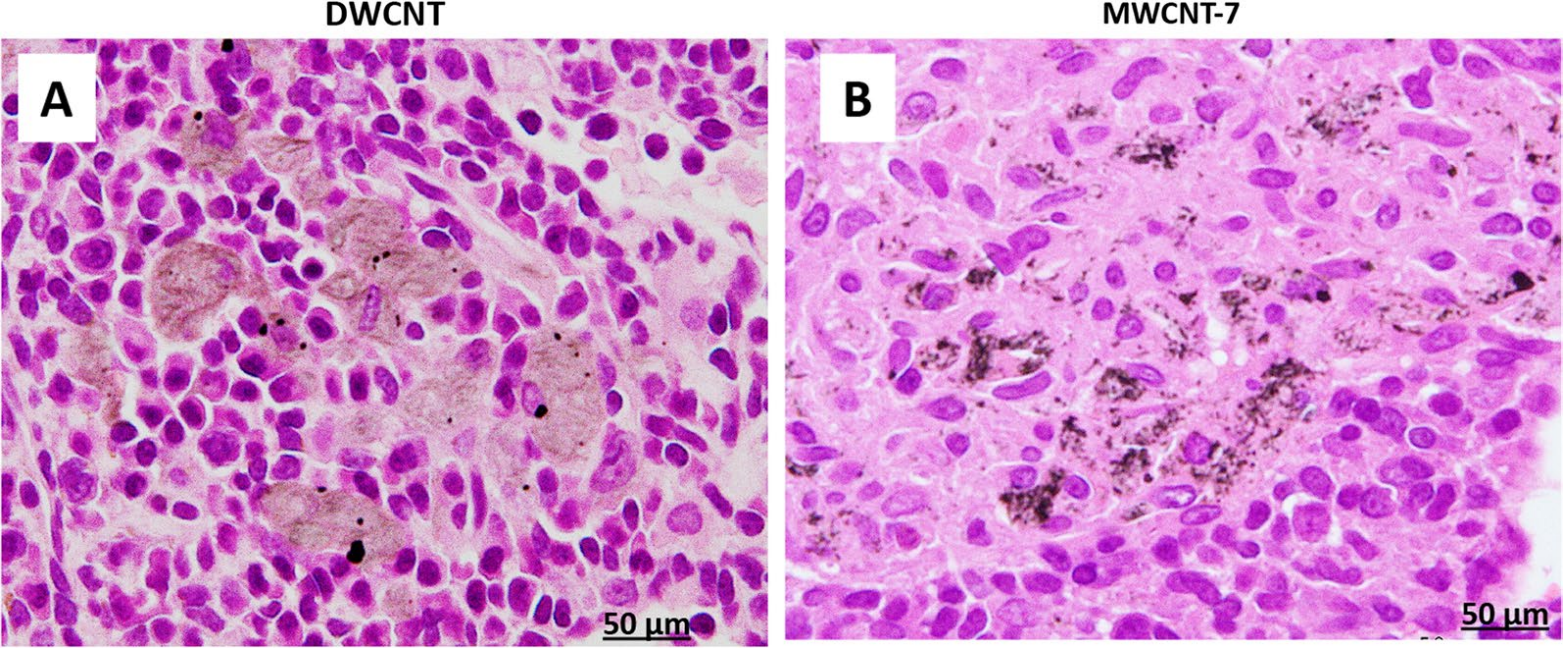
Mediastinal lymph nodes of rats administered 0.5 mg DWCNT (**A**) or 0.5 mg MWCNT-7 (**B**). Both fibers are shown to translocate from the alveoli to the mediastinal lymph nodes

Table 4 shows pathological parameters at 52 weeks and Table 5 shows pathological parameters at the final sacrifice. Histopathological observation at 52 weeks and at the final sacrifice showed that DWCNTs were mostly phagocytosed by groups of macrophages forming foreign body granulation tissue (Fig. 3), and at both time points granulation tissue was significantly increased in all treated groups compared to the vehicle group. Granulation tissue was also significantly higher in the DWCNT 0.25 and 0.5 mg groups compared to the MWCNT-7 group at week 52 and in the DWCNT 0.5 mg group compared to MWCNT-7 group at the final sacrifice. In contrast to DWCNT, MWCNT-7 fibers were commonly observed in free macrophages and some fibers were observed free in the alveolar space (Fig. 3). Both DWCNT and MWCNT-7 fibers were found in the parabronchial and mediastinal lymph nodes (Fig. 4).

**Table 4:** Cell proliferation and inflammation related parameters in the lung tissue at 52 weeks

	Untreated	Vehicle	DWCNT 0.125 mg/rat	DWCNT 0.250 mg/rat	**DWCNT** **0.500 mg/rat**	**MWCNT** **0.500 mg/rat**
Granulation tissue count/cm^2^ mean ± S.D	1.1 ± 0.35	2.4 ± 1.2	98.8 ± 31.0***	189 ± 32.5***^,#^	404.4 ± 88.8***^,#^	65.4 ± 11.8**
Macrophage count/cm^2^ mean ± S.D	1.45 ± 0.23	6.93 ± 1.47	21.72 ± 4.2***	33.01 + 6.4***^,#^	49.1 + 5.79***^,#^	15.88 ± 5.79**
Alveolar cell PCNA index %	10.77 ± 3.00	18.10 ± 8.65	16.51 ± 7.85	17.81 ± 7.40	25.85 ± 4.74	44.52 ± 7.55 **
CCL2 pg/mg lung protein mean ± S.D	3.30 ± 0.28	3.65 ± 0.44	16.69 ± 4.65***	23.36 + 4.33***^,#^	22.37 + 3.7***^,#^	9.49 ± 0.98***
CCL3 pg/mg lung protein mean ± S.D	6.82 ± 0.56	8.19 ± 0.73	40.32 ± 10.4**	69.09 + 7.84***^,#^	135.57 + 11***^,##^	24.86 ± 8.4*

*,**,***Difference from the Vehicle group at p < 0.05, 0.01, 0.001, respectively

^#,##^Significantly increased compared to MWCNT-7 at p < 0.05, 0.01 respectively

**Table 5:** Cell proliferation and inflammation related parameters of the lung tissue at the final sacrifice^a^

	Untreated	Vehicle	DWCNT 0.125 mg/rat	DWCNT 0.250 mg/rat	DWCNT 0.500 mg/rat	MWCNT^a^ 0.500 mg/rat
Granulation Tissue count/cm^2^ mean ± S.D	0.9 ± 0.3	1.3 ± 0.6	50.9 ± 5.2**	118.9 ± 68.8***	365.1 ± 81.7***^,#^	94.9 ± 35.3**
Macrophage count/cm^2^ mean ± S.D	54.6 ± 16.4	75 ± 23.0	361.8 ± 53.4***	502.8 ± 59.1***^,#^	479.1 ± 48.2***^,#^	314.7 ± 91.6***
Alveolar PCNA index %	3.62 ± 1.7.3	4.48 ± 1.88	8.83 ± 4.28*	18.54 ± 7.35**	21.27 ± 10.57**^#^	14.10 ± 10.29**
Visceral pleural PCNA index %	0.20 ± 0.28	0.36 ± 0.26	0.44 ± 0.17	1.40 ± 1.23	1.32 ± 0.72	15.20 ± 13.22**^$^
Parietal pleural PCNA index %	2.00 ± 0.43	2.30 ± 0.66	2.00 ± 0.712	1.90 ± 0.77	2.60 ± 0.28	23.75 ± 32.56***^$^
Pulmonary Collagen deposition %^b^	13.04	8.50	13	18.15	20.47*	27*
Subpleural Collagen deposition % ^c^	4.20	2.25	5.50	4.45	16.87	64.20***^$^
CCL2 ng/mg lung protein mean ± S.D	0.77 ± 0.3	1.6 ± 0.87	6.67 ± 2.44***	8.40 ± 1.73^***,#^	10.74 ± 1.94^***,##^	5.20 ± 1.6***
CCL3 ng/mg lung protein mean ± S.D	0.58 ± 0.27	0.55 ± 0.16	0.71 ± 0.16^*,#^	0.75 ± 0.21^*,#^	0.74 ± 0.19^*,#^	0.52 ± 0.12

^a^In the MWCNT-7 group, 16 animals died from malignant mesothelioma after 52 weeks and before 91 weeks. Because of the extensive invasion of the lungs by the mesotheliomas, these animals could not be assessed for the parameters shown in Table 5. Therefore, only the values obtained from the nine MWCNT-7 treated rats that survived for 91 weeks are shown in Table 5

^b^Results are expressed as the percentage area of pulmonary collagen deposition/total alveolar tissue area

^c^Results are expressed as the percentage area of subpleural collagen deposition/total pleural tissue area

*,**,***Significantly increased from the Vehicle at p < 0.05, 0.01, 0.001 respectively

^#,##^Significantly increased compared to MWCNT-7 at p < 0.05, 0.01 respectively

^$^Significantly increased in compared to DWCNT groups: p < 0.01

**Fig. 5:**
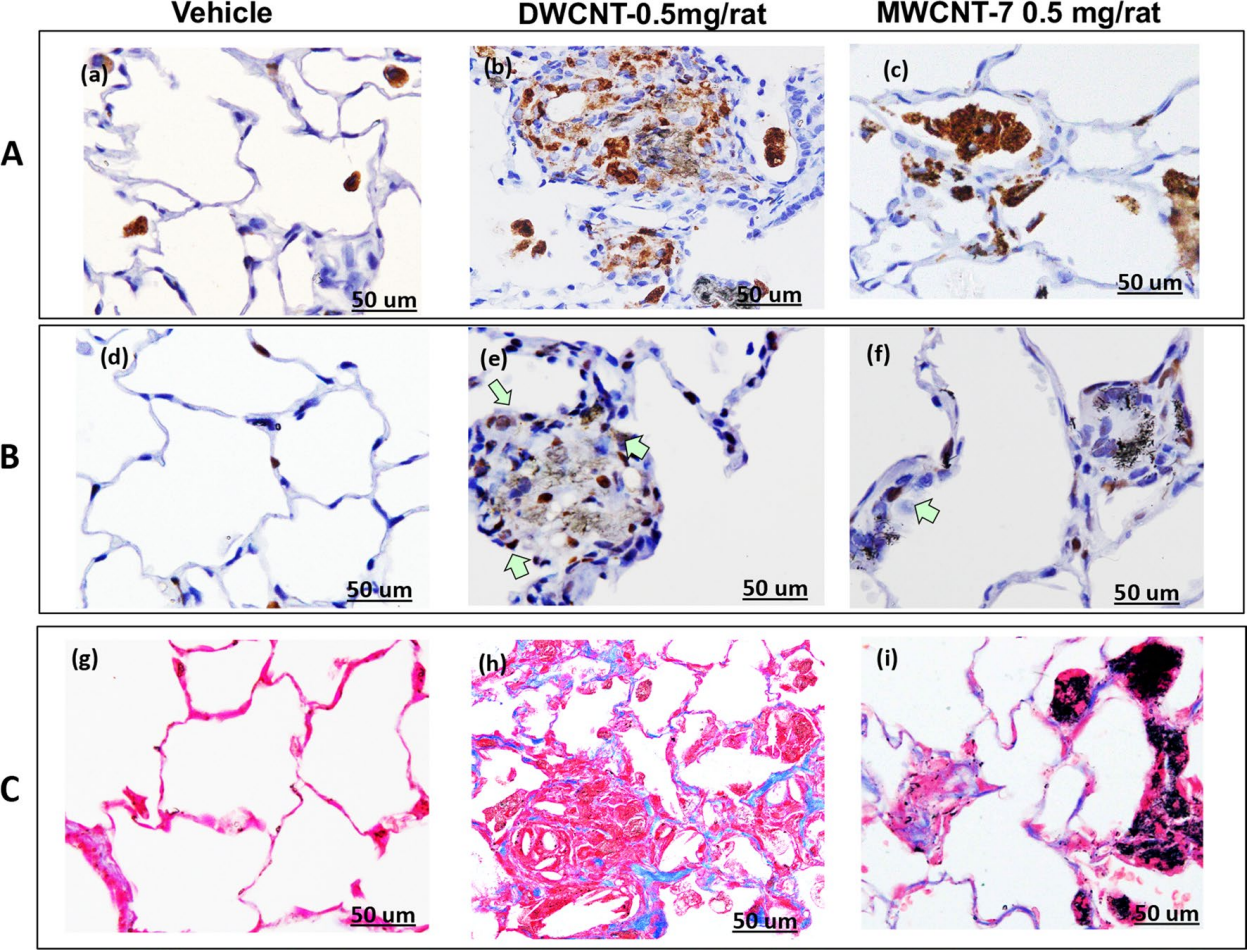
**A** shows CD68 immunostaining of lung tissue from rats in the (**a**) Vehicle, (**b**) 0.5 mg DWCNT, and (**c**) 0.5 mg MWCNT-7 groups at the final sacrifice. **B** shows PCNA immunostaining of the lung tissue from rats in the (**d**) Vehicle, (**e**) 0.5 mg DWCNT and (**f**) 0.5 mg MWCNT-7 0.5 groups at the final sacrifice. **C** shows Masson’s trichrome collagen staining of lung tissue from rats in the (**g**) Vehicle, (**h**) 0.5 mg DWCNT, and (**i**) 0.5 mg MWCNT-7 groups at the final sacrifice

Immunohistochemical evaluation showed that at 52 weeks and at the final sacrifice, the CD68 positive macrophage count was significantly increased in all treated groups compared with the vehicle group (Tables 4 and 5). The macrophage count was also significantly increased in the DWCNT 0.25 and 0.5 mg groups compared to the MWCNT-7 group at both time points. Figure 5A shows CD68 immunostaining of vehicle, 0.5 mg DWCNT, and 0.5 MWCNT-7 lungs at the final sacrifice.

PCNA indices in the lung at 52 weeks are shown in Table 4. PCNA indices of alveolar cells in the rats administered DWCNT were not different from the controls at week 52. In contrast, PCNA indices of alveolar cells in the rats administered MWCNT were significantly increased compared to the controls at week 52.

PCNA indices at the final sacrifice are shown in Table 5 and Fig. 5B shows PCNA immunostaining of vehicle, 0.5 mg DWCNT, and 0.5 MWCNT-7 lungs at the final sacrifice. The PCNA index of alveolar cells was significantly increased in all treated groups compared with the vehicle group. The visceral and parietal pleural mesothelial PCNA indices were not increased in any of the DWCNT groups compared to the controls. The visceral and parietal pleural mesothelial PCNA indices were significantly higher in the MWCNT-7 group compared to the vehicle group and to the DWCNT groups.

Pulmonary collagen deposition at the final sacrifice is shown in Table 5, and Fig. 5C shows Masson's trichrome staining of vehicle, 0.5 mg DWCNT, and 0.5 MWCNT-7 lungs at the final sacrifice. Fibrotic changes with increased deposition of collagen in the alveolar wall, parabronchial areas, and within the granulation tissues were observed in the DWCNT and MWCNT-7 groups. Pulmonary collagen deposition was significantly higher in the 0.5 mg DWCNT and MWCNT-7 groups compared to the vehicle control group.

**Fig. 6:**
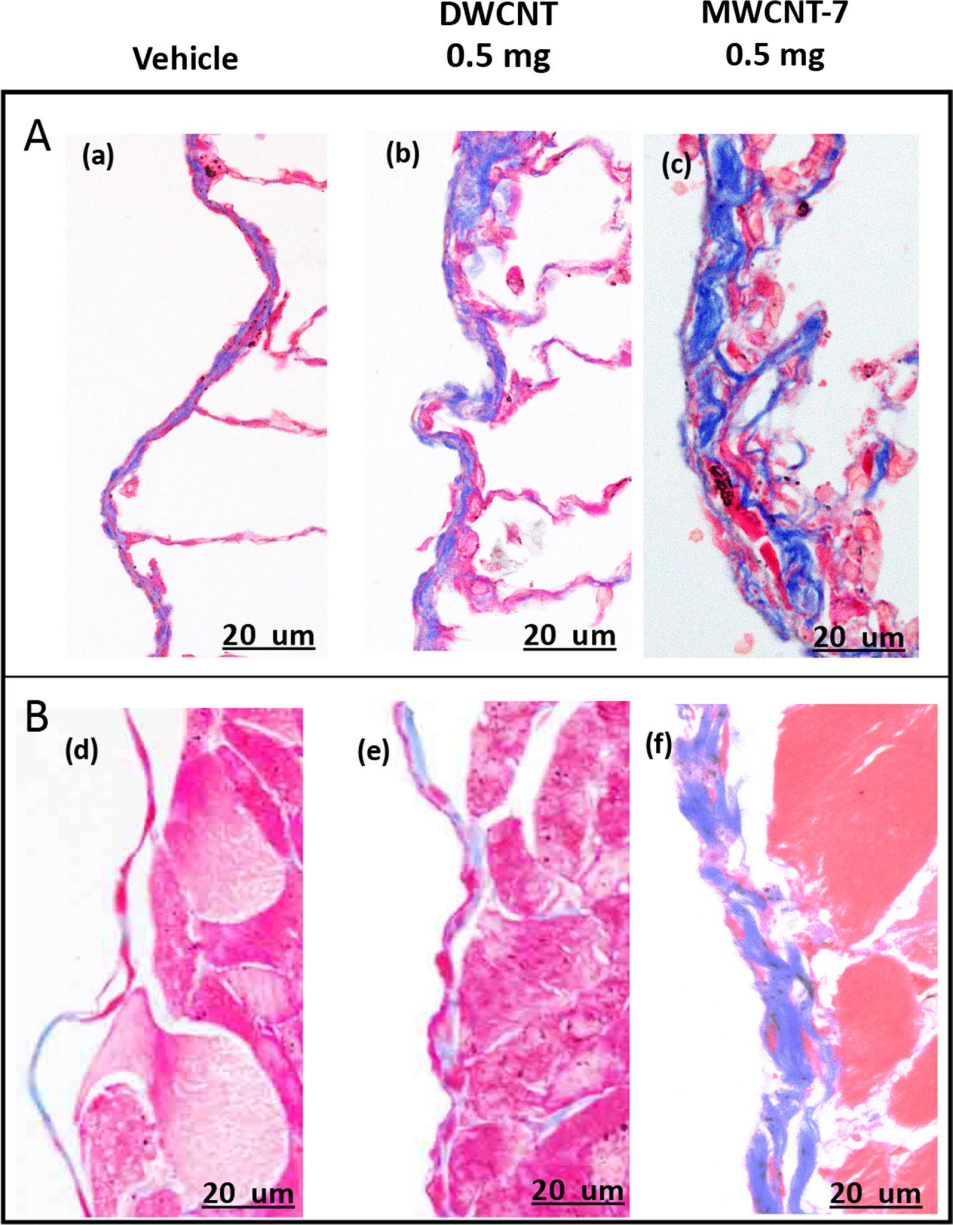
**A** shows Masson’s trichrome collagen staining of the visceral pleura of rats from the (**a**) Vehicle, (**b**) DWCNT 0.5 mg, and (**c**) MWCNT-7 0.5 mg groups at the final sacrifice. Panel **B** shows Masson’s trichrome collagen staining of the parietal pleura of rats from the (**a**) Vehicle, (**b**) DWCNT 0.5 mg, and (**c**) MWCNT-7 0.5 mg groups at the final sacrifice

Subpleural collagen deposition at the final sacrifice is shown in Table 5, and Fig. 6 shows Masson’s trichrome collagen staining of visceral and parietal pleura. Subpleural collagen deposition was somewhat increased in the visceral pleura but not in the parietal pleura of the 0.5 mg DWCNT group, and overall subpleural collagen deposition was not significantly increased in the 0.5 mg DWCNT group compared to the controls. MWCNT-7 treated rats had increased collagen deposition at both the visceral and parietal pleura, and overall subpleural collagen deposition was significantly higher in the MWCNT-7 group than the vehicle controls and the 3 DWCNT groups.

The levels of CCL2 and CCL3 in lung tissue at 52 weeks and the final sacrifice are shown in Tables 4 and 5. The levels of both CCL2 and CCL3 in lung tissue at both 52 weeks and the final sacrifice were significantly higher in all treated groups compared with the vehicle control group. The CCL2 and CCL3 levels in the DWCNT 0.25 and 0.5 mg groups were significantly higher than the MWCNT-7 group at both 52 weeks and at the final sacrifice.

### Lung fiber burden at 52 weeks and the final sacrifice

**Fig. 7:**
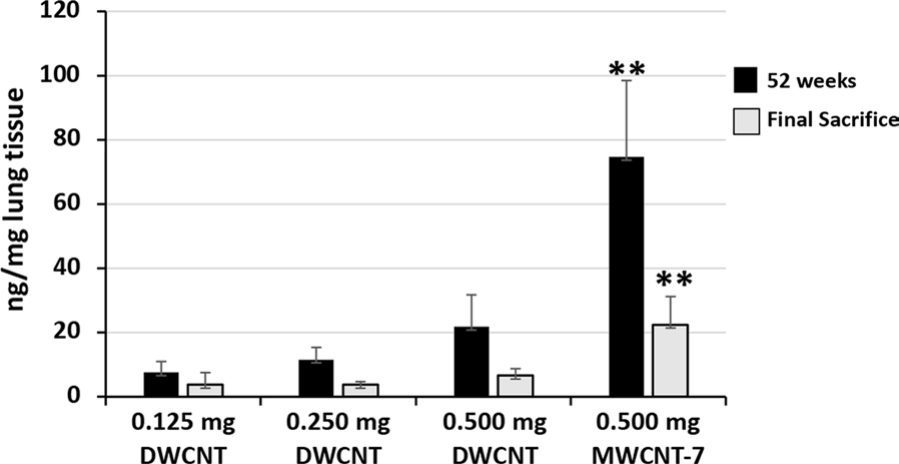
Lung tissue fiber burden at 52 and final sacrifice. **p < 0.01 versus DWCNT (0.5 mg)

The lung fiber burden at 52 weeks and the final sacrifice is shown in Fig. 7. At 52 weeks, the amounts of DWCNTs in the lungs were proportional to the initial dose administrated. Lung fiber burden decreased from week 52 to the final sacrifice, however, more than 1% of the initially instilled fibers remained in the lung 104 weeks and 91 weeks after instillation of DWCNT and MWCNT-7, respectively, and both DWCNT and MWCNT-7 were readily detectable in lung tissue sections at the final sacrifice (see Figs. 3 and 5), indicating that DWCNT as well as MWCNT-7 was biopersistent in the rat lung.

### Electron microscopic observation

**Fig. 8:**
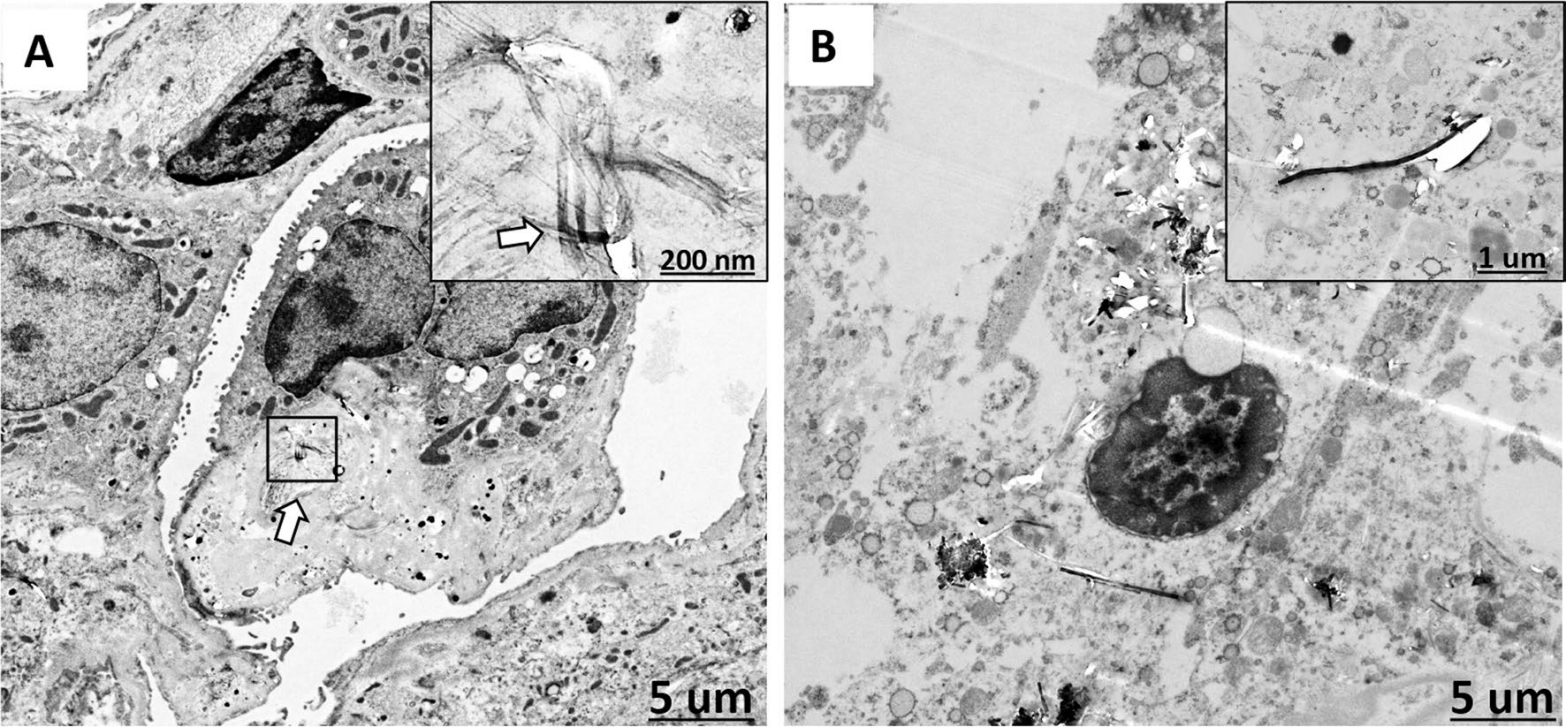
**A** shows a TEM image of a rat lung treated with DWCNT (0.5 mg) showing several very thin curved fibers (arrow and at higher magnification in the boxed area) in a multinucleated giant cell. **B** shows a TEM image of rat lung treated with MWCNT-7 (0.5 mg) showing rigid fibers in the cytoplasm of an alveolar macrophage. Inset shows an enlarged view of an MWCNT-7 fiber in the macrophage cytoplasm

**Fig. 9:**
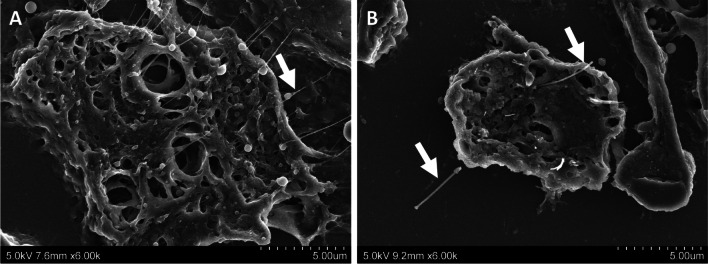
**A** shows an SEM image of numerous thin DWCNT fibers (arrow) engulfed by a macrophage. **B** shows an SEM image of a rigid MWCNT-7 fiber penetrating through the cell membrane of a macrophage (arrow). Free MWCNT-7 fibers were also observed in the alveolar space

At 104 weeks, DWCNT agglomerates were mostly found engulfed by groups of macrophages forming granulation tissue with multinucleated giant cells (Fig. 3). Figure 8 is a TEM image of irregular tangled DWCNT fibers in a multinucleated giant cell. Figure 9 is an SEM image of numerous thin DWNCT fibers engulfed by a single macrophage. In contrast, TEM and SEM images of MWCNT-7 treated rats sacrificed at 91 weeks primarily showed single MWCNT-7 fibers or small bundles of MWCNT-7 fibers associated with macrophages (Figs. 8 and 9). MWCNT-7 fibers were also found free in the alveolar space (Fig. 9). Importantly, MWCNT-7 fibers could also be seen penetrating the macrophage cell membrane (Fig. 9).

### DNA adductomes formation in the lung tissue

Additional file 1: Fig. S1 shows adductome maps of rats at 52 weeks. The relative peak areas and the number of peaks are given for each group. There were no significant differences in either peak area or the number of peaks between any of the groups. No adducts specific to any of the treated groups were found.

### HMBG1 levels in the pleural lavage fluid at the final sacrifice

**Fig. 10:**
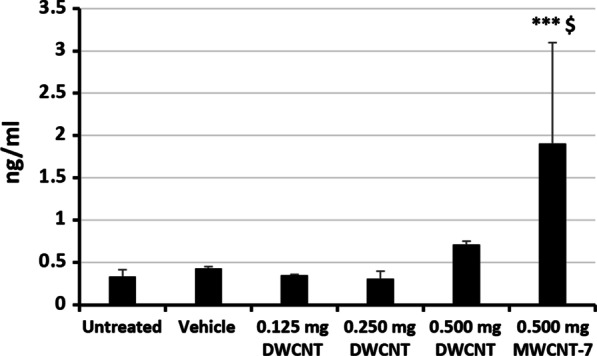
HMBG1 levels in the pleural lavage fluid at the final sacrifice. ***p < 0.001 versus vehicle. ^$^p < 0.05 versus DWCNT (0.5 mg)

HMGB1 is a serum marker for mesothelioma [32–35]. HMGB1 is released from mesothelial cells when they undergo necrosis induced by exposure to asbestos fibers [32, 36], and the released HMGB1 is involved in malignant transformation [34, 36–39]. Therefore, the PLF of rats with malignant mesothelioma is expected to contain elevated levels of HMGB1. At the final sacrifice, the PLF levels of HMGB1 were not elevated in the DWCNT treated rats, but were significantly elevated (p < 0.001) in the MWCNT-7 treated rats (Fig. 10): untreated (0.34 ± 0.08 ng/ml), vehicle (0.43 ± 0.24 ng/ml), 0.125 mg DWCNT (0.35 ± 0.12 ng/ml), 0.25 mg DWCNT (0.30 ± 0.09 ng/ml), 0.5 mg DWCNT (0.71 ± 0.48), and 0.5 mg MWCNT-7 at 91 weeks (1.91 ± 1.19 ng/ml). Notably, HMGB1 levels were not elevated the pleural lavage fluid of the 0.5 mg DWCNT treated rat that developed visceral pleural mesothelioma (0.52 ng/ml); the rat treated with 0.25 mg DWCNT that developed visceral pleural mesothelioma was found dead and therefore pleural lavage fluid could not be collected from this rat. As discussed below, the pleural mesotheliomas that developed in the DWCNT treated rats are unlikely to be relevant to human mesothelioma development.

## Discussion

This is the first long-term, 2-year in vivo study to assess the toxicity and carcinogenicity of DWCNTs in the rat lung. Rats were untreated or administered vehicle or 0.125 mg, 0.25 mg, or 0.5 mg DWCNT per rat or 0.50 mg MWCNT-7 per rat: MWCNT-7 was used as the reference material. We found that DWCNTs were biopersistent in the rat lung and induced chronic inflammation, and that the 0.50 mg group developed lung fibrosis and lung tumors (see Tables 1, 3, and 5). At 52 weeks, rats in the 0.25 mg and 0.50 mg groups had significantly elevated levels of hyperplasia in the lung, and at 104 weeks one rat in the 0.125 mg DWCNT group, 2 rats in the 0.25 mg DWCNT group, and 4 rats in the 0.50 mg DWCNT group had developed adenocarcinomas (see Tables 2 and 3). While these incidences were not statistically significant compared to the vehicle controls, which did not develop adenocarcinomas, the apparent dose response agrees with the possibility that in this study the DWCNT fibers that were tested may have had carcinogenic potential. In addition, the incidence of total lung tumors, bronchiolo-alveolar adenoma and bronchiolo-alveolar carcinoma combined, was significantly higher in the 0.5 mg DWCNT group compared to the vehicle controls (see Table 3). Overall, these results indicate that the possibility that DWCNTs are carcinogenic cannot be ignored and that further long-term studies to assess the in vivo toxicity and carcinogenicity of DWCNT should be carried out.

It is notable that two rats administered DWCNT developed malignant pleural mesothelioma (see Table 3). The development of this tumor is rare in rats: the Japan Bioassay Research Center historical control data shows that approximately 1 of 1000 rats spontaneously develop malignant pleural mesothelioma and a retrospective review by Tokarz et al., 2021, identifies spontaneous primary pleural mesothelioma developing in approximately 1 of 2000 male F344 rats [40]. This suggests that development of these tumors is biologically significant. However, the PLF of the rats administered DWCNT did not have elevated levels of HMGB1. In contrast, the PLF of the rats administered MWCNT-7 did have elevated levels of HMGB1 (see Fig. 10). HMGB1 is released from mesothelial cells when they undergo necrosis induced by exposure to asbestos fibers [32, 36], and the released HMGB1 is involved in malignant transformation [34, 36–39], suggesting that rats developing fiber-associated pleural mesothelioma would be expected to have elevated levels of HMGB1 in their PLF. In addition, HMGB1 is a serum marker for mesothelioma in humans [32–35], suggesting that HMGB1 is also released from cells in patients with advanced mesothelioma. Therefore, the PLF of rats with either developing or advanced malignant pleural mesothelioma would be expected to contain elevated levels of HMGB1, as is the case for the rats administered MWCNT-7. The absence of elevated levels of HMGB1 in the groups administered DWCNT suggests that these lesions were not associated with fibers in the pleural cavity. Another notable point is that the pleural mesotheliomas that developed in the DWCNT administered rats developed in the visceral pleural. Fiber-associated pleural mesotheliomas are induced by fibers that are retained in the pleural cavity. These fibers are retained at the parietal pleura as they are carried by the pleural fluid that flows out of the pleural cavity through the stomata present in the parietal pleura, and consequently, fiber-associated pleural mesotheliomas initially develop in the parietal pleura [41–46]. This also suggests that development of visceral mesothelioma in the DWNCT administered rats was not due to DWCNT fibers present in the pleural cavity. Finally, it is well known that the visceral pleura in rats is thin, consisting of the pleural mesothelial layer and a basement membrane lying directly over the alveoli (see Fig. 6A), while in humans the visceral pleura has substantial submesothelial connective tissue. Consequently, in rats, but not in humans, interactions occurring in the lung alveoli adjacent to the visceral pleura could affect visceral pleural mesothelial cells, making the mechanism of visceral mesothelioma induction in rats not applicable to humans. Therefore, development of pleural mesothelioma in the visceral pleura coupled with the absence of elevated levels of HMGB1 in the PLF of the rats administered DWCNT support the premise that development of pleural mesothelioma in the DWCNT treated rats in the current study is not biologically relevant to humans.

Two rats also developed malignant peritoneal mesothelioma. However, aged male Fischer 344 rats are prone to developing spontaneous peritoneal mesotheliomas [31]. Therefore, the low incidence of these mesotheliomas suggests that they are not treatment related.

Another notable result in the present study is the apparently low incidence of lung tumors in the MWCNT-7 treated rats: 2 rats developed lung carcinomas and 1 rat developed a lung adenoma. However, it needs to be noted that 16 rats in this group died before the end of the study due to mesotheliomas, and therefore, the incidence of lung tumor development in rats surviving to the final sacrifice of this group of animals at 91 weeks (9 rats) was numerically higher than in the DWCNT groups. Notably, an incidence of 2 carcinomas and 3 lung tumors in 9 rats is identical to the incidence of carcinomas and lung tumors in a two year inhalation study by Kasai et al. [17]. In the Kasai et al. study, 11 carcinomas and 16 lung tumors developed in 50 male rats exposed to 2 mg/m^3^ MWCNT-7 for 2 years. A low incidence of lung tumor development (0 rats) accompanied by a high incidence of mesothelioma development was also observed in a previous study in which rats administered MWCNT-7 by TIPS died before the end of the study period due to mesothelioma [20]. Therefore, tumor development in rats treated with MWCNT-7 in the present study was similar to that reported previously.

The DWCNT fibers used in the present study formed tangled agglomerates: agglomerate is used as defined by Walter 2013 [47]. Agglomerate formation is a known characteristic of CNTs [48, 49]. In the atmosphere of the working environment, CNTs are usually found as aggregates or agglomerates and rarely as single fibers [2, 50]. DWCNT agglomerates deposited beyond the ciliated airways were not completely moved out of the lung or broken down by alveolar macrophages (see Table 1 and Fig. 7). It is well known that persistence of CNTs in the lung induces inflammation [2, 48, 49, 51, 52]. In our earlier study, at 6 weeks after administration of DWCNT to rats mild inflammation was observed and granulomas, which are an inflammatory sequestration response [53, 54], containing DWCNT fibers had developed [30]. In the present study, biopersistence of the DWCNT fibers in the rat lung induced persistent inflammation: at 52 and 104 weeks macrophage count, CCL2 and CCL3 expression, and granuloma formation was significantly elevated in the lungs of all DWCNT treated rats (Tables 4 and 5). Chronic inflammation is a hallmark of cancer [55–58] and at 52 weeks hyperplasia was observed in the lungs of the 0.25 and 0.50 mg DWCNT treated rats and at 104 weeks the alveolar PCNA index was elevated in all DWCNT treated rats and tumor incidence was significantly increased the rats administered 0.5 mg DWCNT.

Fibrosis is characterized by the excess accumulation of the extracellular matrix (ECM) in response to non-resolving chronic inflammation [51, 52, 59–61]. Pulmonary fibrosis can impair lung function resulting in morbidity and mortality in humans [51, 59, 62]. During the chronic response to biopersistent CNTs, granulomas containing CNTs and characterized by local accumulation of activated macrophages serve as fibrogenic foci [59]. In our study, at 52 and 104 weeks all three groups of DWCNT treated rats had significant amounts of granulation tissue, increased macrophage counts, and elevated levels of CCL2 and CCL3, indicating the presence of activated macrophages, in the lung. Figure 5 shows collagen deposition at the fibrogenic foci formed by granulomas containing DWCNT and MWCNT-7 fibers. At 104 weeks elevated collagen deposition in the lung was observed in rats administered 0.25 and 0.50 mg DWCNT, and in the rats administered 0.50 mg DWCNT collagen deposition was significantly higher than the vehicle controls. In addition, fibrosis can be associated with tumorigenesis [52, 63], and in our study, the rats administered 0.50 mg DWCNT had significantly elevated collagen deposition and a significantly increased tumor incidence.

An important point regarding the results of the present study is how DWCNT can be tumorigenic in the rat lung but thin tangled CNTs do not induce the development of mesotheliomas, even when injected into the peritoneal cavity at relatively high levels [18, 19, 21]. One likely factor is the reported role of HMGB1 in the development of mesotheliomas [34, 36–39]. Interaction of rigid fibers with mesothelial cells can result in cytotoxicity and release of HMGB1 from the cell, and the released HMGB1 can promote malignant transformation [34, 36–39]. In contrast, thin tangled CNTs are reported to be less cytotoxic to mesothelial cells [64], and consequently, thin tangled fibers could be considerably less carcinogenic to mesothelium than rigid fibers.

As discussed above, the DWCNT fibers used in this study displayed characteristics typical of biopersistent fibers. As it is highly likely that individual DWCNT fibers would be phagocytosed and removed from the lung by alveolar macrophages, the biopersistence and subsequent induction of chromic inflammation, fibrosis, and tumorigenesis were the result of the agglomerates formed by the fibers. Since CNTs are typically found as aggregates of agglomerates and not as single fibers in the workplace [2], agglomerate formation by the DWCNT fibers used in the present study is not unexpected. Therefore, this characteristic must be taken into consideration when assessing the toxicity of CNTs, especially thin CNTs.

Occupational exposure to CNTs rage from less than 1 μg/m^3^ to several μg/m^3^ [65–68]. Using an exposure of 1 μg/m^3^, and an alveolar surface area of 0.4 m^2^ for a Fisher 344 rat and 102 m^2^ for a human [69] and a 7% deposition rate in humans (Figs. 6–6 & 6–7 [70]) a human would have to inhale 1,820 mg DWCNT to acquire the same DWCNT burden as a rat instilled with 0.5 mg DWCNT. Given a minute ventilation rate of 20 L/min for a human doing light work (https://doi.org/10.1016/0273-2300(92)90040-G), in an environment containing 1 μg/m^3^ DWCNT, 1,820,000 μg DWCNT would be inhaled by an unprotected human worker in approximately 91,050,000 min or approximately 1,517,500 h. This is obviously much longer than a working lifetime. However, the lung toxicities of fibers differs in rats and humans. For asbestos, the only type of fiber for which a direct comparison can be made for lung toxicities of rats and humans, the lung tissue burden of crocidolite asbestos that caused a carcinogenic response in rats (1250 fg/μg dry lung tissue weight) was 6000 times higher than in humans (0.2 fg/μg dry lung tissue weight) [71, 72]: it is reasonable that rats have stronger lung defenses to dusts than humans as rats are exposed to dusts much more than humans and short-lived rats will be exposed to fibers for a much shorter period than long-lived humans allowing ras to tolerate much higher fiber loads than humans. This difference in the carcinogenic response of rats and humans to respired fibers suggests that it is possible for a worker to inhale a potentially toxic level of DWCNT in the workplace. Notably, the 2013 report by the national Institute for Occupational safety and Health Current Intelligence Bulletin 65 states that given the known adverse respiratory effects of CNTs in animals, all types of CNT and CNF (carbon nanofibers) should be considered an occupational respiratory hazard (see Sect. 4 Conclusions—Hazard and Exposure Assessment in [73]).

One limitation of the present study is that poorly soluble particles (PSPs) of low toxicity can have toxic effects in the rat lung. Toxic effects occur when pulmonary macrophage clearance of the particles is impaired due to an excessive particle load in the lung: impaired clearance due to an excessive particle load is known as particle overload and the condition is known as lung overload. However, instillation of 0.5 mg PSPs into the rat lung does not result in an excessive particle load in the lung. For example, administration of 0.5 mg of rutile-type nano TiO_2_ (r-nTiO_2_) particles by TIPS did not result in particle overload in the rat lung: rats administered 0.25 mg and 0.5 mg r-nTiO_2_ by TIPS did not develop any lesions or clinical signs related to the instilled particles [74]. However, in that study the r-nTiO_2_ was cleared from the lungs while in the present study, the DWCNT fibers were not cleared from the lungs. Therefore, it is possible that biopersistence of DWCNT in the rat lung could be viewed as particle overload. Another point concerning particle overload is that the mechanism associated with the toxic effects mediated by particle overload are chronic inflammation, fibrosis, and tumorigenesis [75], and this mechanism is the same mechanism by which fibers generate toxic effects in the lung (summarized in Fig. 4.2 in [10]). One proposal regarding particle overload in rats and humans is that in humans, but not rats, particles are transferred into interstitial sites where they do not interact with macrophages, thereby averting chronic inflammation, fibrosis, and tumorigenesis (Fig. 4 in [75]). However, interstitialisation of PSPs may not apply to DWCNT agglomerates. A defining characteristic of particle overload induced tumorigenesis is that it is generally specific to rats [75]. Therefore, to further investigate the toxicity and carcinogenicity of DWCNT in the lung, toxicity and carcinogenicity of DWCNT needs to be assessed using a second test animal, such as mice, [76]: Notably, IARC accepts evidence of tumorigenicity in two animal species as sufficient evidence of carcinogenicity in experimental animals [77].

The present study has two other limitations. While we show that the possibility that respired DWCNT is a toxic to humans cannot be ignored, our study does not have unambiguous evidence that the DWCNT fibers we tested are carcinogenic in rats. However, after demonstrating the possibility that DWCNT fibers are toxic to the rat lung, a future study with a larger number of animals in the DWCNT groups can be performed. The third limitation is that administration of a test substance by instillation can be used for hazard identification and hazard ranking, but instillation cannot be used for risk characterization [78]. Therefore, if DWCNTs are identified as toxic in experimental animals, inhalation studies to characterize DWCNT risk need to be done.

Importantly, the response of the rat lung to particle overload should not be interpreted as indicating that the rat is not suitable for use in inhalation studies that test the toxicity of respirable particles. As stated by Bos et al., 2019, "Unless available data clearly point out otherwise, rat pulmonary toxicity including lung inflammation and tumour formation, needs to be considered relevant for human hazard and risk assessment" [79].

A final point regarding this study is how instillation studies using rats compare with inhalation studies. Instillation bypasses the upper respiratory tract, allowing pulmonary deposition of test material without nasal filtering, and instillation delivers a high amount of the test material to the lungs as a single bolus. Not unexpectedly, a primary difference between instillation and inflammation is that acute effects, such as acute inflammation, are higher in rats administered test substances by instillation compared to rats exposed to test substances by inhalation [80–83]. Another difference is that exposure by inhalation results in build up of the inspired material throughout the period of exposure. In contrast, administration by instillation results in the test material being deposited in the lung at the beginning of the study, allowing material-associated processes to proceed for the entire study period (Sect. 3.4 in [84]). In the case of MWCNT-7 induced mesothelioma, in the inhalation study conduced by the Japan Bioassay Research Center, at the end of the 2 year inhalation exposure to 2 mg/m^3^ MWCNT-7 resulted in accumulation of 1.8 mg fibers in the lungs of male rats and 1468 fibers in the pleural area and mesothelial hyperplasia [17], while in the study by Numano et al. 2019 [20], instillation of 1.5 mg MWCNT-7 per rat, which is approximately the same level of MWCNT-7 that accumulated in the lungs of male rats exposed to 2 mg/m^3^ MWCNT-7 by inhalation, the rats developed mesotheliomas. As argued by the authors, this suggests that if the study by JBRC could have been extended by an additional 12–18 months, inhalation exposure to MWCNT-7 would have resulted in the development of mesothelioma. However, most studies that compare the effects of administration by instillation and inhalation conclude that the toxicity of test substances administered by these two methods are similar. Two studies directly compared the pulmonary toxicity of MWCNTs administered by instillation and inhalation, and both studies found that both methods of administration resulted in similar pulmonary toxicities [85, 86]. Several other studies that administered nanoparticles using instillation and inhalation also found both methods resulted in similar pulmonary toxicities [81, 82, 87–92]. Importantly, instillation of 2 mg potassium hexatitanate or 1 mg zinc oxide nanoparticles or 0.5 mg rutile type nano-TiO2 per rat did not result in pulmonary toxicity [74, 93, 94], indicating that reasonable amounts of poorly soluble materials can be instilled into the rat lung without inducing particle overload associated pulmonary toxicity. Therefore, as stated by Oberdörster and Kuhlbusch, 2018, instillation studies are appropriate for hazard identification and hazard ranking, although instillation studies are not appropriate for hazard characterization [78].

## Conclusions

This is the first 2-year study to assess the toxicity and carcinogenicity of DWCNTs after administration into the rat lung. DWCNT did not induce toxic or carcinogenic effects in the pleural cavity, which is in agreement with long-term 2-year studies that found that rigid but not thin tangled CNTs induced mesothelioma when injected into the peritoneal cavity [18, 19, 21]. However, DWCNT administered at a dose of 0.5 mg/rat did cause pulmonary fibrosis and lung tumor development. This was most likely due to agglomerate formation by the fibers, resulting in biopersistence of the fibers in the lung and consequent chronic pulmonary inflammation which in turn promoted pulmonary fibrosis and tumor development. These results indicate that assessment of the toxicity of thin CNTs should not be based solely on the physiochemical characteristics of single fibers. In addition, the results of this study indicate that the possibility that DWCNTs may be toxic to humans cannot be ignored.

## Methods

### Nanomaterials

Two types of CNT were used in this study. DWCNT (brand name Tocana; Toray Industries,Inc., Tokyo, Japan) with an iron content below detectable limits and water content equal to 0.025% by weight (information provided by the company) and MWCNT-7 (Mitsui Chemicals Inc., Tokyo, Japan) with an iron content of 0.3% by weight [95]. Table 6 shows the water and iron content of the fibers.

**Table 6:** Iron and water content of DWCNT and MWCNT-7 before dosing

	Iron content (wt%)	Water content (wt%)
DWCNT	0^a^	0.025^a^
MWCNT-7	0.3^b^	NA ^c^

^a^Information provided by Toray the manufacturer company

^b^Sakamoto et al. 2018[95]

^c^Not available

### Animals

Nine-week old male F344 rats were purchased from Charles River Japan Inc. (Yukohama, Japan). The animals were housed in the Center for Experimental Animal Science of Nagoya City University Medical School, maintained on a 12 h light–dark cycle, and received Oriental MF basal diet (Oriental Yeast Co., Tokyo, Japan) and tap water ad libitum. The experimental protocol was approved by the Animal Care and Use Committee of Nagoya City University Medical School, and the research was conducted according to the Guidelines for the Care and Use of Laboratory Animals of Nagoya City University. The experiment was started after a 2-week acclimation and quarantine period.

### Experimental design

A total of 180 rats 11 weeks old were divided into six groups: Group 1 (26 rats) no treatment; Group 2 (30 rats) vehicle (saline with 0.5% Pluronic F-68: Sigma-Aldrich Merck); Group 3 (30 rats) DWCNT (0.125 mg/rat); Group 4 (33 rats) DWCNT (0.25 mg/rat); Group 5 (30 rats) DWCNT (0.5 mg/rat); and Group 6 (30 rats) MWCNT-7 (0.5 mg/rat): MWCNT-7 was used as the reference material. One rat in group 1 died before the 52 week interim sacrifice and was removed from the study, therefore, group 1 had 26 rats. Rats were administered the test solutions by TIPS as previously described [74]. Briefly, rats were anesthetized with 3% isoflurane and administered 0.5 ml vehicle or test material suspensions (15.6 μg, 31.25, or 62.5 μg of test material in 0.5 ml vehicle) using a micro sprayer (series IA–1B Intratracheal Aerosolizer Penn-century, Philadelphia, PA). Rats were administered one dose every other day over a 15-day period (8 administrations for total doses of 0.125, 0.25, or 0.5 mg DWCNT per rat and 0.5 mg MWCNT per rat). The amount of DWCNT administered to the rats was approximately equivalent to or less than the doses used in the studies by Crouzier et al., Tian et al., Sager et al., and El-Gazzar et al. [26–28, 30]. The dose of MWCNT-7 was one third the amount of MWCNT-7 that caused mesothelioma in rats in a previous study [20]. At week 52 an interim sacrifice was performed on 5 rats from the Untreated and Vehicle groups, 5 rats from the DWCNT 0.125 mg/rat group, 7 rats from the DWCNT 0.25 mg/rat group, 6 rats from the DWCNT-0.5 mg/rat group, and 5 rats from the MWCNT-7 group. All animals found moribund after 52 weeks underwent terminal sacrifice. Because of the loss of 16 animals by the end of week 90 in the MWCNT-7 group, final terminal sacrifice of the remaining 9 animals in this group was performed at week 91. Final terminal sacrifice of the untreated, vehicle, and DWCNT treated rats was at week 104. Final sacrifice refers to the terminal sacrifices performed at weeks 91 and 104.

### Preparation of the test materials

Test materials were weighed and dispersed in tert-butyl alcohol by sonication for 10 min and stored frozen at −20 °C. Shortly before administration, the T-butyl alcohol was removed using an Eyela Freeze Drying machine (FDU-2110; Tokyo Rikakikai Co., Ltd., Tokyo, Japan), and the DWCNT and MWCNT-7 were suspended in saline containing 0.5% Pluronic F-68 (PF68, Sigma-Aldrich Merck) at 31.25, 62.5, and 125 μg/ml. After suspension in Saline + PF68, test materials were sonicated for 2 min four times at 3000 rpm using a polytron PT 1600E bench top homogenizer (Kinematika AG, Lattau, Switzerland). Immediately prior to administration, the suspensions were sonicated for 30 min using a Tomy Ultrasonic disruptor, UD-211, equipped with a TP-040 micro tip (Tomy Seiko Co., Ltd., Tokyo, Japan) at a power setting of 4.

### Characterization of the test materials before and after TIPS administration

After sonication, described above, and before administration to the rats' lungs, 20 μl of each test material suspension was placed on a micro grid membrane pasting copper mesh (EMS 200-Cu, Nisshin EM Co., Ltd., Tokyo, Japan) for measurement of DWCNT and MWCNT-7 prior to TIPS administration. For measurement of DWCNT and MWCNT-7 in the lungs of rats at 52 and at the final sacrifice weeks after instillation about 1 gm of paraformaldehyde fixed lung tissue was digested as previously described [96]. Briefly, tissues were incubated in Clean 99-K200® (Clean chemical Co., Ltd., Ibraki, Japan) overnight or until complete dissolution of lung tissues. The digested solution was then centrifuged at 12,000 rpm for 30 min and the supernatant was discarded. The pellet was resuspended in distilled water and sonicated by a short burst (approximately 2–3 s) from a microson ultrasonic cell disruptor (Misonix Incorporated).. The pellet was collected by centrifugation and washed two more times. After a final centrifugation, the pellet was resuspended in 200 μl of distilled water and the specimens were collected on EMD MilliporeTM Polycarbonate Membrane Filters (Millipore, Tokyo, Japan). Fibers were viewed by SEM (Field Emission Scanning Electronic Microscope; Hitachi High Technologies, Tokyo, Japan) at 5–10 kV and TEM (Transmission electron microscope; EDAX, Tokyo, Japan) at 15–50 K. Photos were analyzed by NIH image analyzer software (NIH, Bethesda, Maryland, USA). At least 200–300 fibers of each type of CNT were measured.

### In vitro biodegradation

DWCNT and MWCNT-7 fibers were added to cultures of RAW cells, a mouse macrophage cell line, and to plates without cells (control). Cultures were incubated overnight with the CNTs at 37 °C in a humidified incubator. The cells were washed twice with PBS to remove materials not associated with the cells. Control plates were not handled. The cultures were then incubated for an addition 4 days. After 5 days incubation with CNTs, culture media was removed from the RAW cell cultures, and the cells were washed twice with PBS to remove materials not associated with the cells. The cells were then harvested and the CNTs recovered from the cells. For the control plates, the CNTs were collected from the cell-free culture media. The collected CNTs were measured using far-infrared absorption to estimate the effective CNT length [97].

### Electron microscopic viewing of fibers in lung tissues

For high magnification viewing, H&E stained slides were immersed in xylene to remove the cover glass, dried, and processed for SEM (Model S Field Emission SEM; Hitachi High Technologies, Tokyo, Japan). For ultrafine viewing, a small piece of paraformaldehyde fixed lung tissue was imbedded in epoxy resin and processed for TEM (EDAX, Tokyo, Japan).

### Measurement of DWCNT and MWCNTs in the lung

Measurement of the amount of CNT fibers in the lung tissue was performed as described previously [23, 96].

### Tissue sample collection and histopathological examination

At necropsy, blood samples were collected via the abdominal aorta under deep isoflurane anesthesia and serum samples were stored at − 80 °C. Organs, including lung, liver, kidney, spleen, brain, heart, and testes were examined for any macroscopic lesions. The trachea, esophagus, lymph nodes (including mediastinal lymph nodes), diaphragm including the diaphragmatic region of the parietal pleura were examined macroscopically and then processed and examined histopathologically.

The 4 right lobes of the lung of each rat were excised at necropsy, frozen in liquid nitrogen, and sored at − 80 degrees for further biochemical analysis. The remaining left lung was inflated and fixed with 4% paraformaldehyde solution in phosphate-buffered saline (PBS) adjusted to pH 7.3 and processed for light microscopic examination. H&E stained tissue sections were evaluated by two board-certified Pathologists of the Japanese society of Toxicologic pathology, Drs. Hiroyuki Tsuda and Satoru Takahashi, and diagnosis of hyperplasia, adenoma, adenocarcinoma, and mesothelioma was done according to the INHAND criteria [98].

PCNA staining of deparaffinized slides processed for antigen retrieval and blockade of endogenous peroxidase activity was performed as previously described [99]. For each lung specimen more than 1000 pulmonary epithelial cells and more than 500 visceral pleural and parietal pleural mesothelial cells were counted blindly in random fields. All nuclei showing brown staining of more than half of the nucleus were considered to be positive.

To determine the degree of inflammation the number of macrophages per cm^2^ of lung tissue was determined: Deparaffinized slides processed for antigen retrieval and blockade of endogenous peroxidase activity were incubated with PBS containing 5% BSA and 5% goat serum for 1 h, then incubated with the macrophage marker anti-CD68 (BMA Biomedicals, August, Switzerland) diluted 1:2000 in PBS containing 1% BSA and 1% goat serum over- night at 4 °C. After overnight incubation, the slides were incubated with secondary antibody (Nichirei Biosciences, Tokyo, Japan) for 1 h, visualized with DAB (Nichirei Bio- sciences, Tokyo, Japan), and counterstained with hematoxylin. Light microscopic images representing at least one cm^2^ from each lung were used to determine the density of the macrophages in the lungs.

Collagen deposition in lung tissues and visceral and partial pleura was quantified in light microscopic images of lung tissues and pleural sections stained with Masson’s Trichrome (Abcam, Tokyo, Japan) using NIH image analyzer software (NIH, Besthesda, Meryland, USA): ten individual images were captured from 2 lung and 3 diaphragm sections from 3 rats per study group. Thresholding using pre-defined RGB criteria for collagen deposition was performed. This allowed collagen to be differentiated from alveolar tissue or pleural tissue. The surface area of fibrosed tissue was measured. Total lung alveolar and pleural tissue surface areas were measured individually by thresholding using predefined RGB criteria for lung alveolar and pleural tissues. The results are expressed as the percentage area of pulmonary collagen deposition per tissue surface area.

### HMGB1 ELISA

At the final necropsy pleural lavage fluid collection was done for all rats as previously described [99]. High mobility group box protein 1 (HMGB1) was measured using a rat HMGB1 ELISA kit (Arigo Biolaboratories; ARG81310) according to the manufacturer's instructions.

### CCL2 and CCL3 ELISA

Frozen right lung tissue samples (approximately 100 mg) were thawed and rinsed 3 times with ice-cold PBS and homogenized in 1 mL tissue protein extraction reagent (Thermo Scientific, Rockford, IL, USA) containing 1% (v/v) protease inhibitor cocktail (Sigma-Aldrich Merck). The homogenates were centrifuged at12000 g for 5 min at 4 °C. Protein content of the supernatant was measured using the BCA Protein Assay Kit (Pierce Biotech). The levels of CCL2 and CCL3 in the supernatant were measured using a Rat MCP-1/CCL2 ELISA Kit (Sigma-Aldrich Merck; RAB0058) and a CCL3 ELISA Kit (LSBio; LS-F5526) according to the manufacturers' instructions.

### Detection of DNA adductomes in the lung tissue

Rat lung DNA was extracted by Gentra® Puregene cell and tissue kit (Qiagen). The DNA (50 μg) was digested by incubation at 37 °C for 12 h in 300 μl of 5 mM Tris–HCl (pH7.4) containing 50 units of DNaseI, 1 unit of Nuclease P1, 2 units of alkaline phosphatase, and 0.225 units of phosphodiesterase. After digestion, internal standards (100 pmol of 2′,3′-dideoxyinosine and 2′,3′-dideoxyadenosine) were added to the DNA hydrolysates. The hydrolysates were filtered through Amicon Ultra 3 kDa centrifugal filters (Sigma-Aldrich Merck; Z677094), and 500 μl of methanol was added to the purified samples. After centrifugation and removal of the methanol, residual methanol was removed by evaporated *in vacuo*. DNA residues were dissolved in 100 μl of 50% methanol, and 10 μl of sample was subjected to LC/MS.

UHPLC-TOF–MS analyses was performed with a Shimadzu UHPLC Nexera X2 system (Shimadzu) using a Synergi Hydro-RP column (2.5 μm, 100 mm × 2 mm, Phenomenex) and Triple TOF 5600 + (SCIEX) with an electrospray ionization device running in the positive ion mode. The detector conditions were as follows: ion spray voltage at 5500 V, source temperature of 350 °C, ion source gas 1, 60 psi, ion source gas 2 60 psi, declustering potential 80 V, collision energies of 45 V, and collision energy spread 15 V. Nitrogen was used as the collision gas. DNA adducts were detected using the MRM^HR^ mode. This strategy was designed to detect the neutral loss of 2′-deoxyribose from positively ionized 2′-deoxynucleoside adducts by monitoring the samples with [M + H]^+^ → [M + H-116]^+^ transitions[100]. In the mobile phases used for LC-TOF–MS analyses, solvent A consisted of a 0.1% (v/v) solution of formic acid in water and solvent B consisted of a 0.1% (v/v) solution of formic acid in acetonitrile. The DNA adducts were eluted from the column using a linear gradient, which started at 95% solvent A and 5% solvent B, and progressed to 100% solvent B over a period of 10 min. The system was then eluted with 100% solvent B for 10 min before being returned to the initial conditions over a period of 10 min to allow for the equilibration of the column. The system was operated at a constant flow rate of 0.2 ml/min for all of the analyses.

### Statistical analysis

Survival rates were analyzed using the Kaplan–Meier method. The incidences of bronchioloalveolar hyperplasia, bronchioloalveolar adenoma, bronchioloalveolar carcinoma, and total lung tumor incidences were analyzed for difference from vehicle controls using GraphPad's Fisher’s extract test (one-sided for comparison of treated rats to the vehicle controls and two-sided for comparison of DWCNT and MWCNT treated rats), and continuous data was analyzed using GraphPad's QuickCals t-Test Calculator. All data are expressed as mean ± standard deviation. p-values < 0.05 were considered to be significant.

## Supplementary Information

**Additional file 1: Figure S1.** Adductome maps of rats at 52 weeks. The relative peak areas and the number of peaks are given for each group. There were no significant differences in either peak area or the number of peaks between any of the groups. No adducts specific to any of the treated groups were found.

## Data Availability

All data generated or analysed during this study are included in this published article and its supplementary information files.
