# Supplementary material for: Assessment of the toxicity and carcinogenicity of double-walled carbon nanotubes in the rat lung after intratracheal instillation: a two-year study
Source: Part Fibre Toxicol. 2022 Apr 22;19:30. doi: 10.1186/s12989-022-00469-8 (PMC9026941; doi:10.1186/s12989-022-00469-8)
Supplement: Supplementary file 1 — Additional file 1: Figure S1. Adductome maps of rats at 52 weeks. The relative peak areas and the number of peaks are given for each group. There were no significant differences in either peak area or the number of peaks between any of the groups. No adducts specific to any of the treated groups were found. [file 12989_2022_469_MOESM1_ESM.pdf]

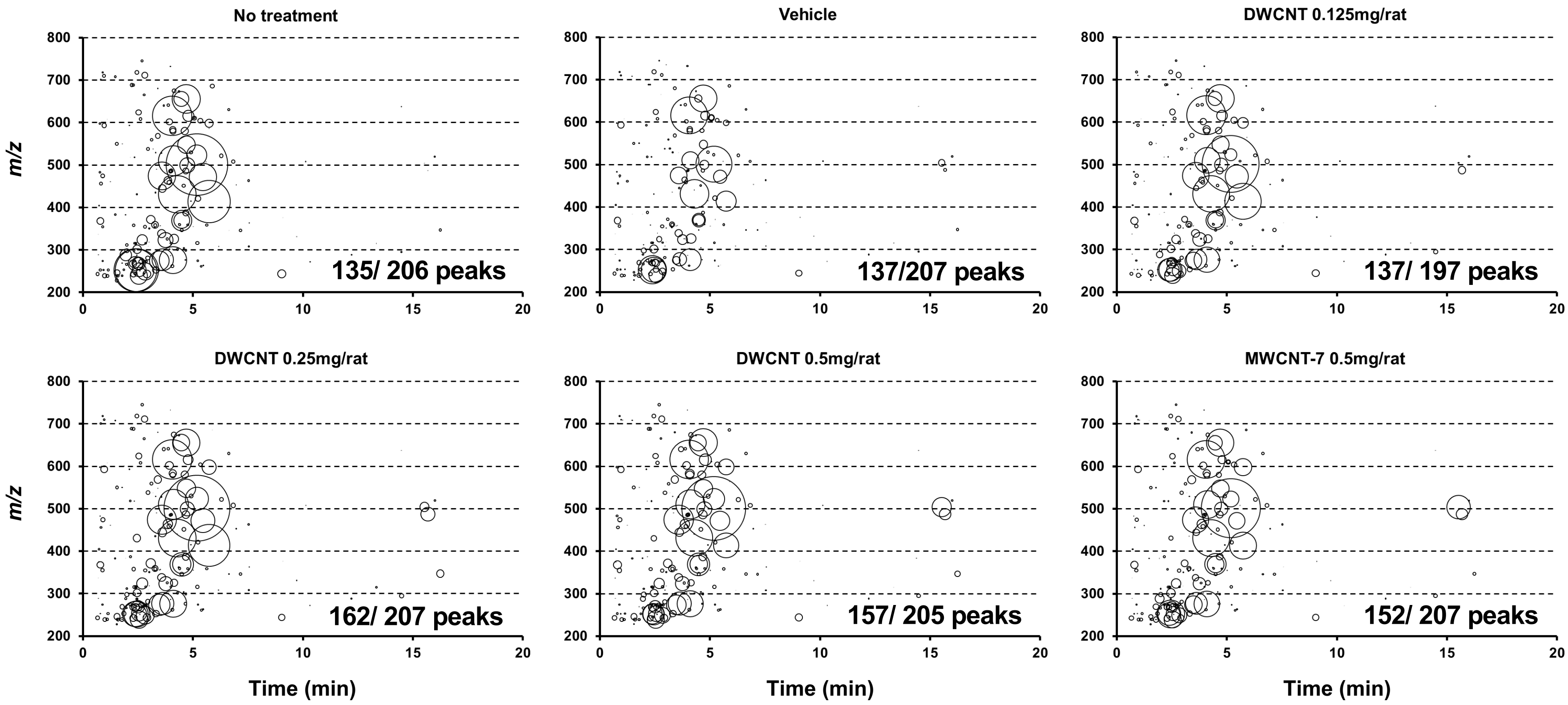

Bubble size = Peak area

Figure S1: Adductome maps of rats at 52 weeks. The relative peak areas and the number of peaks are given for each group. There were no significant differences in either peak area or the number of peaks between any of the groups. No adducts specific to any of the treated groups were found.
